# First isolation of *Arcanobacterium pinnipediorum* from a grey seal pup (*Halichoerus grypus*) in the UK

**DOI:** 10.1007/s12223-021-00932-7

**Published:** 2021-11-26

**Authors:** Mazen Alssahen, Geoffrey Foster, Abdulwahed Ahmed Hassan, Jörg Rau, Christoph Lämmler, Ellen Prenger-Berninghoff, Tobias Eisenberg, Mathew Robinson, Amir Abdulmawjood

**Affiliations:** 1 Institut Für Hygiene Und Infektionskrankheiten Der Tiere, Justus-Liebig-Universität Giessen, Frankfurter Straße 85-91, Giessen, 35392 Germany; 2 SRUC Veterinary Services, 10 Inverness Campus, Inverness, An Lochran, IV2 5NA UK; 3 Department of Veterinary Public Health (DVPH), College of Veterinary Medicine, Mosul University, Mosul, Iraq; 4 Chemisches Und Veterinäruntersuchungsamt Stuttgart (CVUAS), Schaflandstraße 3/2, Fellbach, 70736 Germany; 5 Landesbetrieb Hessisches Landeslabor, Giessen, Schubertstraße 60, Giessen, 35392 Germany; 6 Axiom Veterinary Laboratories, Manor House, Brunel Road, Newton Abbot, TQ12 4PB UK; 7 Institute of Food Quality and Food Safety, Research Center for Emerging Infections and Zoonoses (RIZ), University of Veterinary Medicine Hannover, Bischofsholer Damm 15, Hannover, 30173 Germany

## Abstract

In the present study, a single *Arcanobacterium* (*A*.) *pinnipediorum* strain isolated from discharge of a jaw swelling of a grey seal pup (*Halichoerus grypus*) in England, UK, was identified. This strain was further characterized by phenotypical investigations, by matrix-assisted laser desorption/ionization time-of-flight mass spectrometry (MALDI-TOF MS), by Fourier transform infrared spectroscopy (FT-IR), and genotypically by sequencing the 16S rRNA gene and the genes *gap* encoding glyceraldehyde 3-phosphate dehydrogenase, *tuf* encoding elongation factor tu, and *rpoB* encoding the β subunit of bacterial RNA polymerase. The present study gives a first detailed characterization of the species *A*. *pinnipediorum* from a grey seal in the UK. However, the route of infection of the grey seal with the bacterial pathogen remains unclear.

## Introduction

The genus *Arcanobacterium* (*A.*) was described by Collins et al. (1982) as a group of facultative anaerobic, asporogenous Gram-positive rods. According to Yassin et al. (2011) genus *Arcanobacterium* consists of four species, namely, *A. haemolyticum*, *A. phocae*, *A. pluranimalium*, and *A. hippocoleae*. In the following years, *A. canis* (Hijazin et al. 2012b), *A. phocisimile* (Hijazin et al. 2013), *A. pinnipediorum* (Sammra et al. 2015), *A. wilhelmae* (Sammra et al. 2017), *A. urinimassiliense* (Diop et al. 2017), *A. ihumii* (Fall et al. 2019), and *A. bovis* (Sammra et al. 2020) were described as novel species of this genus. The type strain *A. pinnipediorum* DSM 2710^ T^ was originally isolated in 2004 during a monitoring program from an anal swab of a female harbor seal and characterized phenotypically, by cell wall analysis and genotypically (Sammra et al. 2015, 2018). To the best of our knowledge, no other *A. pinnipediorum* strains have been described elsewhere. The present study was designed to characterize a single *A. pinnipediorum* strain, which represents the first isolation of this bacterial species in the UK and the first from a grey seal (*Halichoerus grypus*).

## Materials and methods

The bacterial strains investigated in the present study included *A. pinnipediorum* 014418, type strain *A. pinnipediorum* DSM 28752^ T^, originally isolated in the German North Sea, the type strains *A. phocae* DSM 10002^ T^ and *A. phocisimile* DSM 26142^ T^, and other type strains of genus *Arcanobacterium* (Hijazin et al. 2012b, 2013; Sammra et al. 2015). *A. pinnipediorum* 014418 was isolated in mixed culture from a swollen mandible discharge of an 8-month-old male grey seal pup in a rehabilitation centre in England. Upon submission, the seal had a high temperature, infected teeth, and gums with discharging pus around the teeth. The animal was treated with amoxicillin/clavulanic acid and clindamycin for 17 days and the discharge resolved. Six weeks later a swab was collected from the bilateral anterior mandible that was swelling and discharging leading to the isolation of *A. pinnipediorum* 014418. The animal was treated again with amoxicillin/clavulanic acid and clindamycin for 25 days. In addition, chin became a hard swelling but there was no discharge. The animal was released 10 days later.

The bacterial culturing of *A. pinnipediorum* 014418 was carried out on 5% sheep blood agar for 48 h at 37 °C under microaerobic conditions. The strain was investigated phenotypically by determination of hemolysis and by VITEK2-compact system (bioMérieux, Nürtingen, Germany) according to the instructions of the manufacturer. The *A. pinnipediorum* strain was additionally analyzed by matrix-assisted laser desorption ionization-time of flight mass spectrometry (MALDI TOF MS, Bruker Biotyper database 8.468, Bruker Daltonik, Bremen, Germany) (Sammra et al. 2018; Wickhorst et al. 2019) and by Fourier transform infrared spectroscopy (FT-IR, Bruker Tensor with HTS-XT, Bruker Optik, Ettlingen, Germany), (Nagib et al. 2014; Sammra et al. 2018).

The presence of *A. phocae* phocaelysin encoding gene *phl* was determined with a previously described loop-mediated isothermal amplification (LAMP) assay. This was performed using a real-time fluorometer (Genie II®, OptiGene, Horsham, UK) (Abdulmawjood et al. 2016; Alssahen et al. 2018).

*A. pinnipediorum* 014418 was additionally investigated by amplification and sequencing of 16S rRNA gene (Hassan et al. 2009; Sammra et al. 2014b, 2015). The DNA template was extracted from freshly cultivated bacterial colonies using the DNeasy Blood and Tissue Kit in accordance with the manufacturer’s instructions (Qiagen, Hilden, Germany). The nucleotide sequence of the bacterial 16S rRNA gene was amplified with forward primer 16S rRNA UNI-L (5′-AGA GTT TGA TCA TGG CTC AG-′3) and reverse primer 16S rRNA UNI-R (5′-GTG TGA CGG GCG GTG TGT AC-′3). The amplicon was used for sequencing with primer 16S rRNA-533F (5′-GTG CCA GCM GCC GCG GTA A-′3) and 16S rRNA-907-R (5′-CCG TCA ATT CMT TTG AGT TT-′3) by Eurofins Genomics GmbH (Ebersberg, Germany). The sequences were analyzed using FinchTV (version, 1.4.0), alignment, and phylogenetic analysis by the Clustal W method using DNASTAR Lasergene version 8.0.2 (DNASTAR Inc., Madison, USA). Furthermore, *A. pinnipediorum* 014418 was characterized by sequencing the genes *gap* encoding glyceraldehyde 3-phosphate dehydrogenase, *tuf* encoding elongation factor tu (Wickhorst et al. 2017, 2019; Sammra et al. 2018), and *rpoB* encoding the β subunit of bacterial RNA polymerase (Ülbegi-Mohyla et al. 2010; Sammra et al. 2013, 2018). The oligonucleotide primer sequences and PCR conditions of the target genes used in the present study are summarized in Table 1.

**Table 1:** Oligonucleotide primer sequences and PCR conditions of the target genes used in the present study

Oligonucleotide primer	Sequence	Expected size of PCR product (bp)	Program*
16S rRNA UNI-L 16S rRNA UNI-R (amplification primer)	5′-AGA GTT TGA TCA TGG CTC AG-′3 5′-GTG TGA CGG GCG GTG TGT AC-′3	1403	1
16S rRNA-533-F 16S rRNA-907-R (sequencing primer)	5′-GTG CCA GCM GCC GCG GTA A-′3 5′-CCG TCA ATT CMT TTG AGT TT-′3	—	—
Gap-F Gap-R	5′-TCG AAG TTG TTG CAG TTA ACG A-3′ 5′-CCA TTC GTT GTC GTA CCA AG-3′	830	2
Tuf-F Tuf-R	5′-GGA CGG TAG TTG GAG AAG AAT GG-3′ 5′-CCA GGT TGA TAA CGC TCC AGA AGA-3′	796	3
RpoB-F RpoB-R	5′-CGW ATG AAC ATY GGB CAG GT-3′ 5′-TCC ATY TCR CCR AAR CGC TG-3′	406	4

^*^1: × 1 (95 °C, 600 s), × 30 (95 °C, 30 s, 58 °C, 60 s, 72 °C, 60 s), and × 1 (72 °C, 420 s). 2: × 1 (94 °C, 180 s), × 30 (94 °C, 30 s, 50 °C, 40 s, 72 °C, 60 s), and × 1 (72 °C, 300 s). 3: × 1 (94 °C, 180 s), × 30 (94 °C, 45 s, 57 °C, 40 s, 72 °C, 60 s), and × 1 (72 °C, 420 s). 4: × 1 (95 °C, 600 s), × 35 (94 °C, 30 s, 50 °C, 30 s, 72 °C, 120 s), and × 1 (72 °C, 600 s)

## Results and discussion

**Fig. 1:**
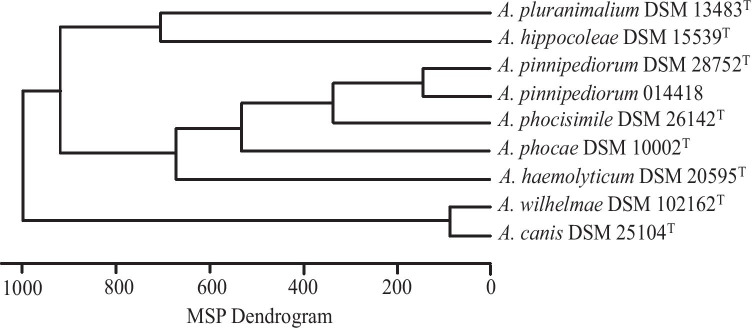
Dendrogram of MALDI-TOF MS main spectra of *A. pinnipediorum* 014418 investigated in the present study in comparison with type strain *A. pinnipediorum* DSM 28752^ T^ and other species of genus *Arcanobacterium*. The MALDI-TOF MS analysis was performed using MALDI Biotyper Version (4.0)

*A. pinnipediorum* 014418 investigated in the present study displayed a relatively small zone of complete hemolysis on 5% sheep blood agar plates and could be classified biochemically using VITEK2-compact system. The biochemical properties of *A. pinnipediorum* 014418 mainly corresponded to properties of *A. pinnipediorum* DSM 28752^ T^. However, differences were observed in a positive D-galactose reaction and negative beta-D-fucosidase reaction of *A. pinnipediorum* 014418 (Table 2). Strain *A. pinnipediorum* 014418 could additionally be identified to the species level by MALDI-TOF MS analysis with the current Bruker database (MBT 8468 MSP library). *A. pinnipediorum* 014418 displayed a close relation to the type strain *A. pinnipediorum* DSM 28752^ T^ and could be clearly separated from other species of genus *Arcanobacterium*. A typical dendrogram of the MALDI-TOF mass spectrum analysis of *A. pinnipediorum* 014418 and various other species of genus *Arcanobacterium* is shown in Fig. 1. MALDI-TOF MS has already been shown to be a rapid and reliable technique for identification of bacteria of genus *Arcanobacterium* and *Trueperella* (Hijazin et al. 2012a; Wickhorst et al. 2017), also including *A. phocae* (Alssahen et al. 2018), *A. phocisimile* (Sammra et al. 2014a), and *A. pinnipediorum* (Sammra et al. 2018) of seal origin. MALDI TOF mass-spectra of *A. pinnipediorum* 014418 and other species of genus *Arcanobacterium* are available by exchange via the MALDI-TOF user platform (https://www.maldi-up.ua-bw.de; Rau et al. 2016).

**Table 2:** Biochemical properties of *A. pinnipediorum* 014418 of the present study and type strain *A. pinnipediorum* DSM 28752^ T^ using VITEK2-compact system

Test*	*A. pinnipediorum* 014418	*A. pinnipediorum* DSM 27852^ T^
D-GALACTOSE	+	−
D-CELLOBIOSE	−	−
SACCHAROSE/SUCROSE	−	−
BETA-GALACTOPYRANOSIDASE Indoxyl	+	+
MALTOTRIOSE	−	−
PHOSPHATASE	−	−
Leucine-ARYLAMIDASE	+	+
Tyrosine-ARYLAMIDASE	+	+
ARGININ-GP	−	−
ALPHA-ARABINOSIDASE	−	−
AESCULIN-Hydrolyse	−	−
L-ARABINOSE	−	−
ELLLMAN	−	−
Ala-Phe-Pro-ARYLAMIDASE	+	+
N-ACETYL-D-GLUCOSAMIN	+	+
5-Brom-4-Chlor-3-Indoxyl-alpha-Galactoside	−	−
BETA-D-FUCOSIDASE	−	+
d-Ribose 2	−	−
Phenylalanin-ARYLAMIDASE	+	+
D-GLUCOSE	+	+
5-Brom-4-Chlor-3-Indoxyl-beta-Glucoside	−	−
BETA-MANNOSIDASE	−	−
5-Brom-4-Chlor-3-Indoxyl-beta-N-Acetyl-Glucosamide	+	+
Phenylphosphonate	−	−
L-Prolin-ARYLAMIDASE	+	+
D-MANNOSE	−	−
UREASE	−	−
5-Brom-4-Chlor-3-Indoxyl-alpha-Mannoside	−	−
ALPHA-L-ARABINFURANOSIDE	−	−
L-Pyrrolidonyl-ARYLAMIDASE	+	+
D-MALTOSE	−	−
5-Brom-4-Chlor-3-Indoxyl-beta-Glucuronide	−	−
PYRUVAT	−	−
ALPHA-L-FUCOSIDASE	−	−
D-XYLOSE	−	−
Gram-stain	+	+
Morph (Morphology)	−	−
Aero (Aerotolerance)	+	+

^*^Tests of VITEK2-compact (bioMérieux, Nürtingen, Germany). The reactions are shown as follows: +  = positive reaction; −  = negative reaction

**Fig. 2:**
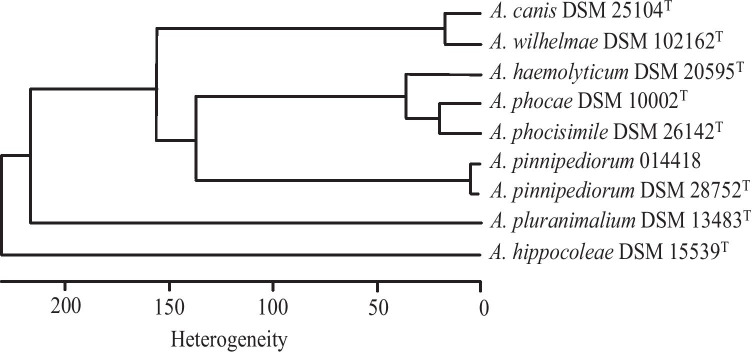
Cluster analysis of infrared spectra of *A. pinnipediorum* 014418 investigated in the present study in comparison with type strain *A. pinnipediorum* DSM 28752^ T^ and other species of genus *Arcanobacterium*. This analysis was performed by using the second derivatives of the spectra (*n* = 2 for each isolate) in the spectral range of 500 to 1400 cm^−1^. Ward’s algorithm was applied in OPUS (version 7.2, Bruker optic, Ettlingen)

FT-IR spectroscopy has already been used as a tool for identification of a large number of clinically relevant pathogens (Kuhm et al. 2009; Samuels et al. 2009; Contzen et al. 2011; Grunert et al. 2013), also including *T. pyogenes* isolated from bovine mastitis (Nagib et al. 2014) and for characterization of *A. pinnipediorum* of seal origin (Sammra et al. 2018). The infrared spectra of *A. pinnipediorum* 014418 of the present study was analyzed by the method described by Nagib et al. (2014). Comparable to the MALDI-TOF MS analysis *A. pinnipediorum* 014418 of the present study yielded a close relation to *A. pinnipediorum* DSM 28752^ T^ and to next closely related members *A. phocae* DSM 10002^ T^, *A. phocisimile* DSM 26142^ T^, and *A. haemolyticum* DSM 20595^ T^ (Fig. 2).

The previously described loop mediated isothermal amplification (LAMP) assay for detection of phocaelysin encoding gene *phl* could be used successfully to identify *A. phocae* of various origins (Alssahen et al. 2018). However, *A. pinnipediorum* 014418 and comparatively investigated *A. pinnipediorum* DSM 28752^ T^ and *A. phocisimile* DSM 26142^ T^ yielded a negative *phl*-reaction indicating the high specificity of this assay for detection of *A. phocae* but not for other species of genus *Arcanobacterium* of seal origin (data not shown).

**Fig. 3:**
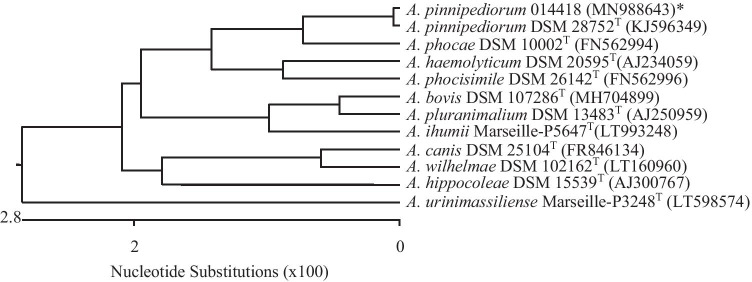
Dendrogram analysis of 16S rRNA gene of *A. pinnipediorum* 014418 investigated in the present study in comparison with the type strain *A. pinnipediorum* DSM 28752^ T^ and type strains of other species of genus *Arcanobacterium* obtained from NCBI GenBank using the Clustal W method of DNASTAR/Lasergene MegAlign program (version 8.0.2). *Accession numbers are given in brackets. ^T^ indicates type strains

Strain *A. pinnipediorum* 014418 of the present study was identified genotypically by sequencing the 16S rRNA gene with a sequence similarity of 99.9% to 16S rRNA gene of type strain *A. pinnipediorum* DSM 28752^ T^ (KJ596349) and a 16S rRNA gene sequence similarity ≤ 98.7% to other species of genus *Arcanobacterium* (Fig. 3).

**Fig. 4:**
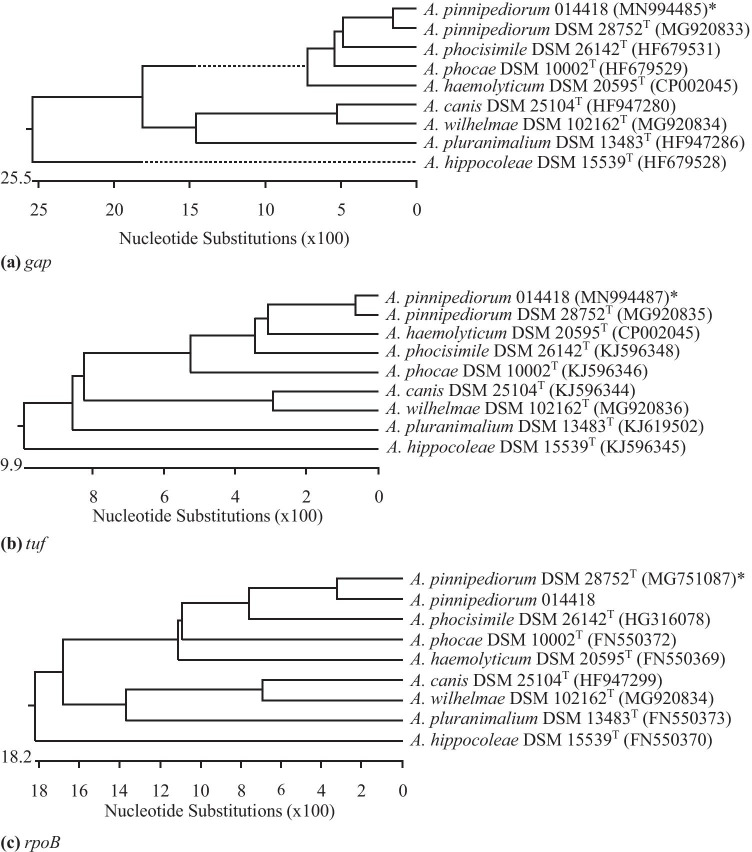
Dendrogram analyses of the genes *gap* **a**, *tuf* **b**, and *rpoB* **c** of *A. pinnipediorum* 014418 investigated in the present study in comparison with the type strain *A. pinnipediorum* DSM 28752^ T^ and type strains of other species of genus *Arcanobacterium* obtained from NCBI GenBank using the Clustal W method of DNASTAR/Lasergene MegAlign program (version 8.0.2). *Accession numbers are given in brackets

According to previous studies *A. phocae*, *A. phocisimile* and *A. pinnipediorum* of seal origin could be further characterized genotypically by sequencing the genomic targets *gap*, *tuf*, and *rpoB* (Sammra et al. 2014a, 2018). Comparable to these studies, *A. pinnipediorum* 014418 showed sequence similarities of the genes *gap*, *tuf*, and *rpoB* of 97.1%, 98.7%, and 93.6%, respectively, to type strain *A. pinnipediorum* DSM 28752^ T^ and sequence similarities of these genes of ≤ 91.2%, ≤ 94.0%, and ≤ 86.5%, respectively, to other species of genus *Arcanobacterium*. A dendrogram analysis of these target genes is shown in Fig. 4a, b, c.

According to the results of the present study, *A. pinnipediorum* 014418 isolated from mandibular discharge of a grey seal pup in England (UK) could successfully be characterized biochemically, by MALDI-TOF MS, by FT-IR spectroscopy, and by sequencing the presented genomic targets. The usefulness of the determination of specific spectra by MALDI-TOF MS and FT-IR analyses and the various genotypic targets for identification of this species have to be further investigated with additional *A. pinnipediorum* strains isolated in other contexts. The biochemical differences observed between *A. pinnipediorum* 014418 and the type strain *A. pinnipediorum* DSM 28752^ T^ might reflect an intra-specific variability caused by evolutionary processes. As already mentioned above, further phenotypical studies and whole genome sequence analyses of *A. pinnipediorum* of various origins will show the unique taxonomic position and the importance of this species in animal infections. However, strain *A. pinnipediorum* 014418 of the present study represented the first isolation of this species in the UK and the first isolation from a grey seal. *A. pinnipediorum* 014418 was isolated from a mixed culture with several other bacteria, including *Enterococcus faecalis*, *Streptococcus lutetiensis*, and a Gram negative cocco-bacillus that could not be identified further, indicating that the pathogenic importance of this species remains to be elucidated. It is perhaps of note, however, that *A. phocae* has often been isolated in mixed infections, including, as is the case in this report, *Streptococcus* spp*.*, from seals in Scotland (G. Foster, unpublished findings). Furthermore, *A. phocae* has been recovered from local lesions including an infected jaw from a mouth lesion of a grey seal (Ramos et al. 1997), providing further similarities with the isolation of *A. pinnipediorum* 014418 from an infected jaw.
